# Neural Stem/Progenitor Cells from the Adult Human Spinal Cord Are Multipotent and Self-Renewing and Differentiate after Transplantation

**DOI:** 10.1371/journal.pone.0027079

**Published:** 2011-11-02

**Authors:** Andrea J. Mothe, Tasneem Zahir, Carlo Santaguida, Douglas Cook, Charles H. Tator

**Affiliations:** Division of Genetics and Development, Toronto Western Research Institute and Krembil Neuroscience Centre, Toronto Western Hospital, Toronto, Ontario, Canada; Université Pierre et Marie Curie-Paris6, INSERM, CNRS, France

## Abstract

Neural stem/progenitor cell (NSPC) transplantation is a promising therapy for spinal cord injury (SCI). However, little is known about NSPC from the adult human spinal cord as a donor source. We demonstrate for the first time that multipotent and self-renewing NSPC can be cultured, passaged and transplanted from the adult human spinal cord of organ transplant donors. Adult human spinal cord NSPC require an adherent substrate for selection and expansion in EGF (epidermal growth factor) and FGF2 (fibroblast growth factor) enriched medium. NSPC as an adherent monolayer can be passaged for at least 9 months and form neurospheres when plated in suspension culture. In EGF/FGF2 culture, NSPC proliferate and primarily express nestin and Sox2, and low levels of markers for differentiating cells. Leukemia inhibitory factor (LIF) promotes NSPC proliferation and significantly enhances GFAP expression in hypoxia. In differentiating conditions in the presence of serum, these NSPC show multipotentiality, expressing markers of neurons, astrocytes, and oligodendrocytes. Dibutyryl cyclic AMP (dbcAMP) significantly enhances neuronal differentiation. We transplanted the multipotent NSPC into SCI rats and show that the xenografts survive, are post-mitotic, and retain the capacity to differentiate into neurons and glia.

Together, these findings reveal that multipotent self-renewing NSPC cultured and passaged from adult human spinal cords of organ transplant donors, respond to exogenous factors that promote selective differentiation, and survive and differentiate after transplantation into the injured spinal cord.

## Introduction

Despite advances in medical and surgical care, current clinical therapies for spinal cord injury (SCI) are limited [1], [2]. One promising approach is cell transplantation to replace damaged cells and promote neuroprotective and neuroregenerative repair. Neural stem cells may be ideal candidates because they can self-renew and are committed to the neural lineage such that they will produce only neuronal and glial cells, and can also be pre-differentiated in vitro along a specific lineage. Neural stem/progenitor cells (NSPC) can be isolated from the developing and adult CNS, and are expanded in vitro with mitogenic growth factors such as EGF and FGF2 [3], [4]. Human embryonic stem cell-derived oligodendrocyte progenitor cells and fetal NSPC are already in Phase 1 clinical trials. The use of adult stem cells avoids the ethical issues of procurement from fetal or embryonic origin. Also, adult stem cells show less oncogenic potential than fetal stem cells or undifferentiated embryonic stem cells [5]–[8]. Spinal cord derived NSPC are region-specific, and thus, may respond more appropriately to the intrinsic micro-environment of the injured spinal cord. Lower vertebrates such as urodele amphibians can regenerate their spinal cord even after full transection. This is in part due to the ependymal cells lining the central canal which proliferate, migrate, and differentiate into neurons and glia and regenerate the spinal cord [9], [10]. Multipotent, self-renewing NSPC can be isolated and cultured from the adult rodent spinal cord when the cultured tissue includes regions of the central canal [11], [12]. We have shown that NSPC generated from the periventricular region of the adult rat spinal cord primarily differentiate into oligodendrocytes in vitro [11] and in vivo [13], [14]. Moreover, we and other groups have shown that transplantation of adult rat spinal cord NPSC into SCI rats promoted functional recovery [15]–[17].

Most experimental SCI studies use NSPC generated from rodents because human stem cells are either not available or are difficult to grow and grow slowly compared to rodent cells [18]. However, considerable species specific differences have been reported [19]–[21]. Human NSPC have been isolated from fetal brain and spinal cord from aborted fetuses [21]–[31] and from adult brain from surgical biopsy specimens and post-mortem tissue [32]–[34]. However, little is known about NSPC from the adult human spinal cord. It has been reported that neurospheres generated from human fetal spinal cord tissue more than 9.5 weeks of gestation cannot be propagated long-term [23], [26], [31]. In 2008, primary neurospheres were generated from the spinal cord of adult human organ transplant donors but these precursors could not be passaged to generate sufficient numbers of cells [35]. To our knowledge, there are no previous reports of successful passaging and transplantation of adult human spinal cord derived NSPC. Thus, this is the first study to show that self-renewing multipotent NSPC can be passaged from adult human spinal cords of organ transplant donors, and to demonstrate that these cells survive and differentiate into both neurons and glia following transplantation into rats with SCI.

## Materials and Methods

### Ethics statement

For the harvesting of human spinal cord tissue, approval was obtained from the University Health Network Research Ethics Board and from the Trillium-Gift of Life Foundation which oversees organ donation in Ontario. All animal procedures were approved by the animal care committee of the University Health Network in accordance with the policies established in the Guide to the Care and Use of Experimental Animals prepared by the Canadian Council on Animal Care.

### Harvesting of spinal cord tissue

Human spinal cord tissue was harvested from adult organ transplant donors after the organs to be transplanted were removed. Spinal cords were harvested from 11 donors, male and female, ranging in age from 2 to 60 years old (Table 1). Generally, one or two 3–6 cm segments of spinal cord from the upper thoracic and/or mid-to-low thoracic levels were excised. The spinal cord segments were removed in the operating room under sterile conditions as soon as possible after cessation of circulation and placed into cold 1x Hanks' Balanced Salt Solution (HBSS) (Gibco-Invitrogen) containing 2% penicillin-streptomycin (Sigma).

**Table 1:** Donors for harvesting of human spinal cord.

Donor	Age	Gender	Time to Harvesting
H1	60	F	7 hrs
H2	52	F	6 hrs
H3	57	M	4 hrs
H4	53	F	3.5 hrs
H5	21	M	7 hrs
H6	50	F	6 hrs
H7	51	F	4 hrs
H8	2	M	3 hrs
H9	16	F	4 hrs
H10	43	M	3.75 hrs
H11	16	M	3 hrs

Summary of donor age, gender, and time to completion of harvesting (time from cardiac cessation to spinal cord harvesting).

### Culture of adult human spinal cord NSPC

The spinal cord was washed in 1x HBSS and the overlying meninges were removed. Using microscissors and jeweller's forceps, the white matter and most of the grey matter were removed leaving the periventricular region including the ependymal, subependymal, and some grey matter tissue surrounding the central canal. The dissected tissue was cut into 1 mm^3^ pieces, enzymatically dissociated in a solution containing 0.01% papain and 0.01% DNase I (Worthington Biochemicals) for 1–2 hours at 37°C, and then mechanically dissociated into a cell suspension. The cell suspension was centrifuged using a discontinuous density gradient consisting of a 10 mg/ml albumin-ovomucoid protein inhibitor (Worthington Biochemicals) to remove cell membrane fragments. Cells were resuspended in serum-free medium (SFM) consisting of Neurobasal-A medium (Gibco-Invitrogen) supplemented with B27 neural supplement (Gibco-Invitrogen), 2 mM L-glutamine (Gibco-Invitrogen), 100 µg/ml penicillin-streptomycin (Gibco-Invitrogen), and hormone mix consisting of 1∶1 DMEM/F-12 (Gibco-Invitrogen), 0.6% glucose (Sigma), 25 µg/ml insulin (Sigma), 100 µg/ml transferrin (Sigma), 5 mM HEPES (Sigma), 3 mM sodium bicarbonate (Sigma), 30 nM sodium seleniate (Sigma), 10 µM putrescine (Sigma), and 20 nM progesterone (Sigma). The SFM was supplemented with 20 ng/ml human recombinant epidermal growth factor (EGF, Sigma), 20 ng/ml human recombinant fibroblast growth factor-2 (FGF2, Sigma), and 2 µg/ml heparin (Sigma). Cells were seeded in the growth factor supplemented medium at a density of 10^5^ cells/well into 6-well culture plates (Nunc) coated with matrigel (BD Biosciences Inc.). We also examined a variety of other adherent substrates such as fibronectin, collagen type I, and poly-D-lysine/laminin (all from BD Biosciences Inc.). These substrates were also found to be effective for adherence of these cells, however, matrigel was used in all the experiments described. One week later, half of the culture medium was replaced with fresh growth factor supplemented SFM twice weekly. One to two weeks later, the culture medium was replaced twice a week with fresh SFM supplemented with growth factors, and cells were subcultured with Accutase before reaching confluence between 4-8 weeks. Cultures were normally incubated at 37°C, 5% CO_2_, 20% O_2_ (normoxia). In initial experiments, we also examined the effects of 10% BIT 9500 serum (StemCell Technologies, Vancouver BC) and 10 ng/ml leukemia inhibitory factor (LIF) (Millipore, Temecula, CA) in growth factor enriched SFM to promote the expansion of NSPC cultured as suspension cultures in uncoated tissue culture flasks. Karyotype analysis was conducted by The Centre for Applied Genomics at the Hospital for Sick Children (Toronto ON).

### Assessment of growth factor dependance, neurosphere formation, effects of LIF and hypoxia

To examine growth factor dependance, the same concentration of cells was plated onto matrigel coated wells in SFM described above in either the absence of growth factors, in 20 ng/ml EGF alone, or in 20 ng/ml FGF2 + 2 µg/ml heparin. Heparin was always added when FGF2 was present since it functions as a cofactor for FGF2. Cells were also cultured as free-floating neurospheres in SFM supplemented with EGF/FGF2. To evaluate the formation of neurospheres, NSPC were seeded at varying densities (<10 cells/µl, 100 cells/µl, 1000 cells/µl, and 10,000 cells/µl) in growth factor supplemented medium in uncoated tissue culture flasks. The flasks were placed back in the incubator and not moved until 7d later when they were examined for neurosphere formation. To examine the effects of LIF, parallel plates were prepared at the time of isolation and 10 ng/ml LIF (Millipore) was added to each well containing the EGF/FGF2 supplemented medium. LIF was replaced with each feeding. To examine the effects of hypoxia, cultures were incubated from the time of isolation in a reduced oxygen (3.5% O_2_, 5% CO_2_) incubator at 37°C. The phenotypic expression pattern of NSPC in proliferating culture conditions was assessed by plating cells or neurospheres onto matrigel coated wells and immunostaining as described below.

### Differentiation of spinal cord NSPC

NSPC were assessed for multipotentiality by plating onto matrigel in SFM with 1% fetal bovine serum (FBS) in the absence of growth factors for 4 weeks to allow the cells to differentiate. To assess the effect of exogenous factors on NSPC differentiation, NSPC were plated in EGF/FGF2 medium at a density of 10^5^ cells/well into 24-well culture plates coated with matrigel. Cultures were incubated for 1 week, and then the medium was replaced with one of the following factors in SFM: 40 and 100 ng/ml PDGF-AA (Peprotech) to promote oligodendrocyte differentiation, and 1 and 4 mM dbcAMP (Sigma-Aldrich) to promote neuronal differentiation. Controls included 1% FBS and SFM. Cultures were incubated at 37°C for an additional 4 weeks and the media changed every week. The phenotypic expression pattern of NSPC in differentiating culture conditions was examined by immunostaining as described below. The total number of cells counted ranged from 180 – 400 cells per marker.

### Immunocytochemistry

Cultures were fixed with 4% paraformaldehyde (PF) in 0.1 M phosphate buffered saline (PBS) and washed with PBS. Cultures were blocked with 10% normal goat serum with 0.3% Triton-X 100 and depending on the antibody with 1.5% bovine serum albumin for 1 hr at room temperature, and then incubated with the primary antibody overnight at 4°C. The following primary antibodies were used: nestin (1∶2000; Millipore, Temecula, CA) for neural stem/progenitor cells; Ki67 (1∶100; Novocastra Laboratories, Newcastle, UK) for proliferating cells; Sox2 (1∶1000; Millipore) a transcription factor for precursor cells; GFAP (1∶2000; Dako, Burlington, ON) for astrocytes; RC1 (1∶1000; Developmental Studies Hybridoma Bank, Iowa City, IA) for radial glia; O4 (1∶1000; Millipore) for oligodendrocyte progenitor cells; CNPase (1∶500; Covance, Emeryville, CA) for oligodendrocytes; βIII-tubulin (1∶2000; Covance) for neuronal progenitor cells; and NF200 (1∶1000; Sigma, St. Louis, MS) for neurons. Cultures were washed with 0.1 M PBS and then incubated with fluorescent Alexa 488 or 568 secondary antibody (1∶500; Invitrogen) for 1 hr, washed with PBS, and incubated in Hoechst (Sigma) to counterstain the nuclei. Species specific non-immune IgG and omission of primary antibody was used as negative controls. In addition, NS1 serum (Developmental Studies Hybridoma Bank) was used as a negative control for RC1 staining. Immunofluorescent staining was examined using a Nikon Eclipse TE 300 microscope.

### Compression SCI and transplantation

Adult female Wistar rats (Charles River, St. Constant, QC, 150-200g) were anesthetized by inhalation of 5% isofluorane which was reduced to 2% during surgery, in combination with a mixture of nitrous oxide and oxygen (1∶2, v/v). With the aid of an operating microscope, the spinal cord was exposed by laminectomy at the T8-T9 vertebral level, and a clip impact-compression injury was made with a 26g force for 1 minute according to the method of Rivlin and Tator [36], a clinically relevant SCI model. One week later, the lesion site was re-exposed, and adult human spinal cord NSPC cultured between 31 to 89 days in vitro were stereotactically injected using a Hamilton syringe with a 32 gauge customized needle and a motorized microinjector (Model 780310; Stoelting, IL). Using the motorized microinjector and operating microscope, a 2.5 µl volume of NSPC containing 250,000 cells was injected 1 mm into the cord at the midline, at 2 sites, 1 mm rostral and caudal to the lesion epicentre. The needle was left in place for an additional two minutes to prevent back-flow of cells. To aid transplant survival and integration, animals were immunosuppressed daily beginning 24 hrs before the day of transplantation until sacrifice with 15 mg/kg of cyclosporine (Sandimmune, Novartis, Dorval, QC, Canada) injected subcutaneously. Rats were sacrificed at 1 wk post-transplantation.

### Tissue processing and immunohistochemistry

Animals were sacrificed with a lethal dose of sodium pentobarbital, followed by transcardial perfusion with 4% PF in 0.1 M PBS, pH 7.4. The rostro-caudal segment of the spinal cord 1–1.5 cm in length encompassing the transplant sites was dissected and the tissue cryoprotected in 30% sucrose. The tissue was embedded in Shandon Cryomatrix compound (VWR Laboratories, Mississauga, ON, Canada), and cryosectioned parasagittally into 20 µm serial sections collected on Superfrost slides (Fisher Scientific, Ottawa, ON, Canada). For immunostaining, sections were rehydrated in 0.1M PBS and permeabilized and blocked with 0.3% Triton-X 100, 10% normal goat serum and 1% BSA for 1 hr at room temperature, and then incubated with the primary antibody overnight at 4°C. The following primary antibodies were used: hMito (1∶100; Millipore) for human specific mitochondrial antigen; hNuc (1∶100; Millipore) for human specific nuclear antigen; Ki67 (1∶100; Novocastra) for proliferating cells; βIII-tubulin (1∶2000; Covance) for neuronal progenitor cells; GFAP (1∶2000; Dako) for astrocytes; and CC1 (1∶1000; Calbiochem, San Diego CA) for oligodendrocytes. For double-labelling, primary antibodies were applied sequentially followed by species-specific fluorescent-conjugated secondary antibodies for 1 hr at room temperature, as described above. Slides were washed with PBS and then coverslipped with Vectashield mounting medium containing DAPI (4′, 6-diamidino-2-phenyl-indole) (Vector Laboratories) to counterstain the nuclei. Species specific non-immune IgG was used as negative controls for each antibody, in addition to tissue controls where available. Immunofluorescent tissue was examined using a Zeiss LSM 510 confocal microscope.

### Quantitative and statistical analysis

To quantify the phenotype of the cultured cells, the number of immunopositive cells for each antibody was counted as a percentage of Hoechst stained cells in 10-15 random fields (n  =  3). Fluorescent cells were examined using a Nikon Eclipse TE 300 microscope, and images were captured using Nikon NIS Elements BR v.3 image acquisition software (R & M Biometrics Inc.; Nashville, TN). To quantify the number of transplanted cells, every eighth section throughout the length of the excised segment of rat spinal cord was analyzed, as we have previously described [13], [14]. All hNuc^+^ cells containing a DAPI^+^ nucleus were counted and the total number of hNuc^+^/DAPI^+^ cells were multiplied by 8 to compensate for the sampling frequency. All data are presented as mean ± standard deviation. Data were analyzed using SigmaStat v.3.11 software (Systat, Point Richmond, CA). One-way analysis of variance (ANOVA) followed by the Bonferroni post-hoc multiple group comparison was used to identify statistical significance among treatments. Statistical significance was determined at the *p<*0.025 level.

## Results

### Adult human spinal cord precursors require an adherent substrate for selection and expansion in EGF/FGF2 culture

**Figure 1:**
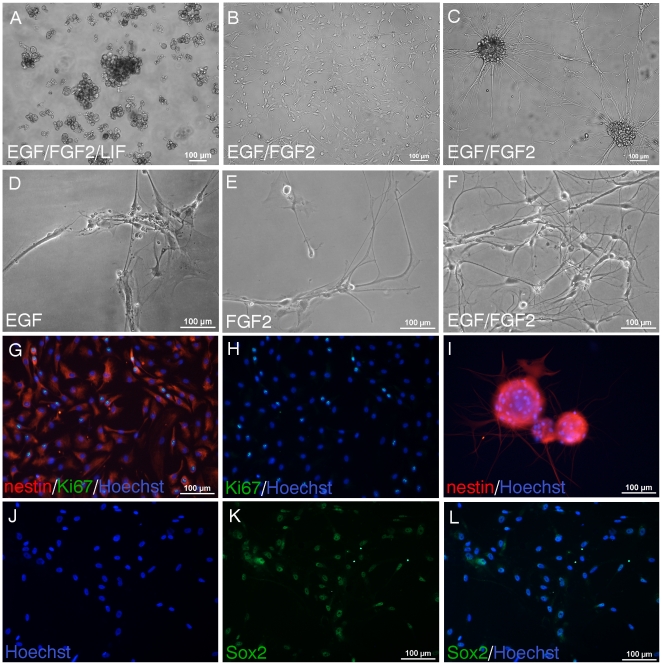
Adult human spinal cord NSPC require an adherent substrate for selection and expansion in EGF/FGF2 culture. A, NSPC were cultured from the time of isolation in uncoated tissue culture flasks. Phase contrast image shows primarily aggregates of debris and some cells at 43div in hypoxic EGF/FGF2 culture containing LIF. B-C, In contrast, in the presence of an adhesive substrate, NSPC are selected in EGF/FGF2 medium and can be expanded continuously. B-C, NSPC cultured and passaged as a monolayer on matrigel for 41div (B) can also form neurospheres in adherent culture (C). D-F, NSPC were cultured for 48div as an adherent monolayer on matrigel. In media containing only EGF (D) or FGF2 (E) fewer cells survived compared to media containing both EGF and FGF2 (F). Both EGF and FGF2 were required for expansion and neurosphere formation. Almost all NSPC expressed nestin (red) (G, 26div). NSPC also expressed Ki67 proliferating nuclear antigen (G, green in overlay; H, green; blue, Hoechst nuclear counterstain). I, Cluster of neurospheres were removed from adherent culture (25div), plated on matrigel coated well in EGF/FGF2 medium, and stained with nestin. Most NSPC expressed Sox2 (66div shown) (K, green; J, corresponding Hoechst staining of nuclei; L, merged).

We harvested spinal cord tissue from 11 organ donors, male and female, 2 to 60 years of age (Table 1). From the first 3 donors, we cultured the cells in suspension culture in growth factor enriched SFM, using methods similar to our previous work with adult rat spinal cord NSPC [11], [13]. Using this approach, most of the cultures consisted of aggregates of cells and debris (Fig. 1A) and rarely, small neurospheres which could not be passaged. We obtained no viable cells after passaging these neurospheres. We attempted to enhance expansion of the suspension cultures with BIT 9500 serum (containing bovine serum albumin, transferrin, and insulin) which was used successfully for the in vitro expansion of hematopoietic stem cell cultures and neonatal human brain progenitor cells [33], [37], [38], but we found no difference. We also examined the effects of LIF, which was important for the self-renewal of mouse embryonic stem cells and increased the rate of proliferation of fetal neural stem cells [29], [39]–[41]. Using a similar concentration of 10 ng/ml LIF, we found no significant differences in the suspension cultures. We also examined the effect of hypoxia by reducing oxygen concentration to 3.5% vs. 20% O_2_ because hypoxia promoted expansion of human neonatal brain NSPC [42], fetal rat brain NSPC [43]–[45], and embryonic mesencephalic neural precursors [44], [45]. However, hypoxia did not increase the number of neurospheres in suspension culture.

It is well known that NPSC can be cultured as free-floating neurospheres in suspension [11], [19], [23], [29], [30], [46]. However, NSPC can also be cultured as an adherent monolayer, which has been used to propagate tissue-specific stem cells without accompanying differentiation [22], [37], [47], [48]. For subsequent isolations, we cultured the adult human spinal cord cells as an adherent monolayer on matrigel coated wells in EGF/FGF2 supplemented medium (Fig. 1B). This strategy was effective for selecting and expanding the NSPC population. Thus, all cultures were viable except for the first 3 isolations which could not be passaged. We also examined several other adhesive substrates including fibronectin, collagen type I, and poly-D-lysine/laminin. These other substrates were also effective for adhesion, however matrigel was used in all the experiments shown. In addition, myelin and debris were removed when the medium was changed during the feedings. As shown in Fig. 1C, sphere-like aggregates of cells also formed from these adherent cultures. Both EGF and FGF2 were required for NSPC expansion and formation of neurospheres (Fig. 1). Fewer cells were present when the medium contained either EGF or FGF2 alone (Fig. 1D-E). Although fewer cells were observed under these conditions, it is possible that the multipotentiality or differentiation capacity of these cells is retained. We generated NSPC as adherent monolayers for at least 9 months involving approximately 10 passages. We generated cells from spinal cord tissue harvested from organ donors if the time from cardiac arrest to culture was 7 hours or less, and we have generated sufficient numbers of NSPC for characterization and transplantation. Karyotype analysis was performed to determine if the genetic stability of the cells was maintained. Cells cultured for 4 months (P5) showed a normal diploid karyotype with no chromosomal abnormalities (data not shown).

### Adult human spinal cord cells express stem/progenitor cell markers in proliferative conditions

In proliferative conditions in EGF/FGF2 culture, almost all human spinal cord cells expressed nestin, a marker for neural precursor cells (Fig. 1G, adherent NSPC; Fig. 1I, neurospheres). These nestin positive cells proliferated in vitro, as shown with Ki67 immunostaining (Fig. 1G, double-labelled for nestin and Ki67; Fig. 1H, corresponding Ki67 staining). Most cells also expressed Sox2 (Fig. 1J–L), a transcription factor essential for embryonic development, maintenance of pluripotency and self-renewal of embryonic stem cells [49], [50]. Also, Sox2 is a persistent marker for multipotential neural stem cells at all stages of development [51], [52].

**Figure 2:**
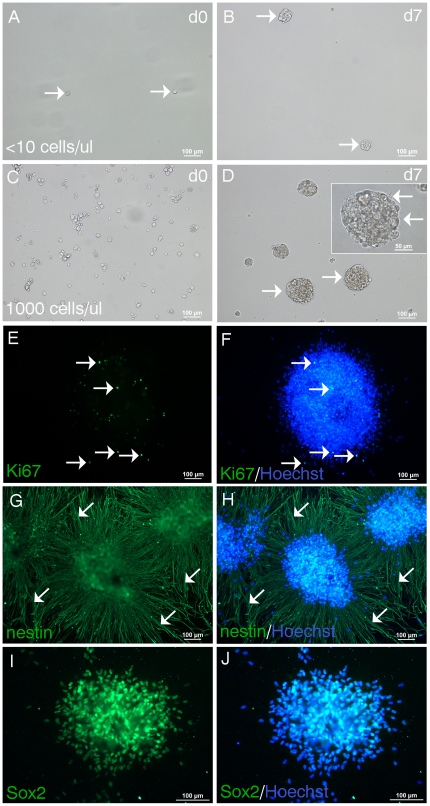
Neurospheres form at clonal density and express stem cell markers. NPSC cultured as an adherent monolayer for 39div were seeded at clonal density (<10 cells/µl) in EGF/FGF2 medium in suspension culture. A, Image taken on the day of seeding (d0) show single cells (arrows). B, 7d later neurospheres have formed (arrows). C, NSPC seeded at higher density (1000 cells/µl) at the time of plating form more neurospheres which are larger (arrows) (D, at 7d). Inset in D shows a high magnification image of a single neurosphere with a phase bright profile and ciliary projections (arrows). Neurospheres were plated onto matrigel and immunostained with stem cell markers. E-F, A single neurosphere showing proliferating Ki67^+^ NSPC (arrows) (E, green) stained with the nuclear dye Hoechst (F, merged). G-H, Three adjacent neurospheres expressing high levels of nestin (G, green) with nestin^+^ processes radially emanating from the neurospheres (arrows) (H, merged). I-J, Most NSPC comprising the neurosphere express Sox2 (I, green; J, merged with Hoechst nuclear counterstain).

To ascertain self-renewal and clonality, cells initially cultured as an adherent monolayer were seeded at clonal conditions [53] in suspension culture supplemented with EGF/FGF2 (Fig. 2A). It has been shown that suspension cultures with low plating densities, as used in the present study, and in the absence of movement during the culture period will result in clonal neurospheres [53]. We found that one week after seeding, small neurospheres formed (Fig. 2B). When cells were seeded at a higher density, more neurospheres formed and were larger (Fig. 2C–D). The formation of larger spheres when cells were seeded at higher densities may be indicative of cell aggregation. The inset in Fig. 2D shows a high magnification image of a single neurosphere with a phase bright profile and typical ciliary projections (Fig. 2D, arrows). These neurospheres could be propagated with passaging by dissociating with Accutase and plating in uncoated tissue culture flasks. Neurospheres were plated onto matrigel and immunostained with Ki67, showing proliferating NPSC (Fig. 2E, F, merged with Hoechst). Human spinal cord neurospheres expressed high levels of nestin, with nestin positive fibers emanating radially from the spheres (Fig. 2G-H, arrows). There was also strong Sox2 immunoreactivity (Fig. 2I–J).

**Figure 3:**
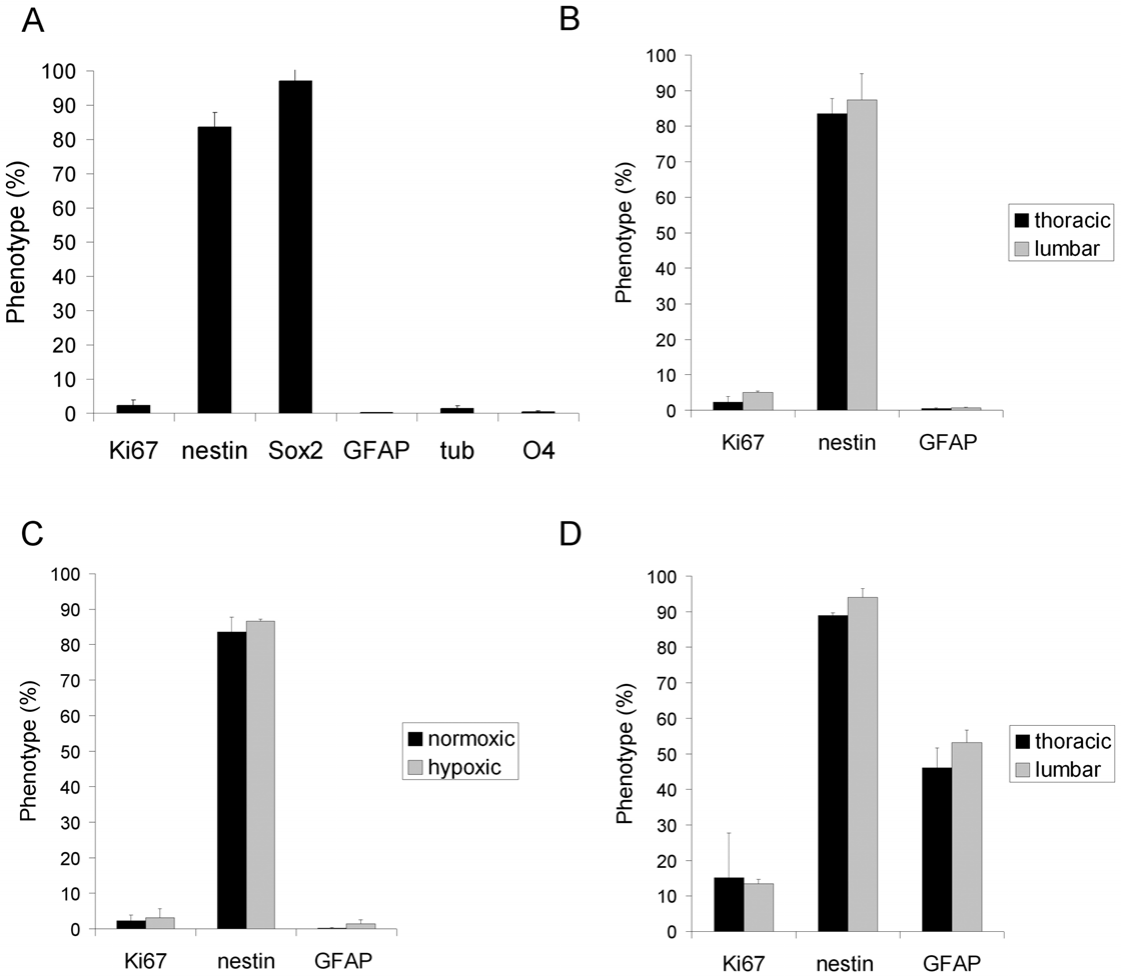
Phenotypic expression profile of human spinal cord NSPC in EGF/FGF2 culture. A, NPSC primarily express nestin and Sox2, and low levels of GFAP, tubulin, and O4. Data shown is averaged from cultured cells generated from the thoracic cord of 3 independent cultures at 35, 66, and 83div. B, NSPC generated from the thoracic and lumbar spinal cord from 2 cultures (34, 66, and 82div) show similar expression levels. C, NSPC cultured in normoxic or hypoxic conditions (2 cultures at 45 and 58div) show no significant differences in Ki67, nestin, and GFAP expression. D, NSPC derived from a younger donor (34 and 45div) also show similar expression levels when cells were generated from the thoracic or lumbar spinal cord. Cultures from the young donor (D) showed significantly higher GFAP expression than cultures from the older donors (B) (p = 0.007).

More than 80% of the human spinal cord cells expressed both nestin and Sox2 (Fig. 3A, nestin 83.6±4.2%, Sox2 97.1±3.4%). 2.3±1.6% of the cells expressed Ki67, and <2% of the cells expressed markers of more differentiated cells, GFAP (0.3±0.07%), βIII-tubulin (1.4±0.7%), and O4 (0.4±0.2%) (Fig. 3A), for astrocytes, neuronal and oligodendrocyte progenitors, respectively. Data shown was averaged from cultured cells generated from the thoracic cord of 3 independent cultures at 35, 66, and 83div. NSPC generated from the thoracic and lumbar spinal cord showed similar expression levels of Ki67, nestin, and GFAP (Fig. 3B and D). Fig. 3B was derived from data averaged from 50 and 51 year old donors (34, 66, and 82div), and Fig. 3D data was averaged from a 2 year old donor (34 and 45div). The most apparent difference was that cultures from the young donor had a higher mitotic index (15.1±12.5% Ki67) than cultures from the older donors (2.3±1.6% Ki67), although this was not statistically significant (p>0.05). Cultures from the young donor showed higher GFAP expression (46.1±5.5%) than the older donors (0.4±0.2% GFAP) (p = 0.007). Cultures in normoxia or hypoxia showed no significant differences in Ki67, GFAP, and nestin expression (Fig. 3C).

### LIF enhances GFAP expression of adult human spinal cord NSPC in EGF/FGF2 culture

**Figure 4:**
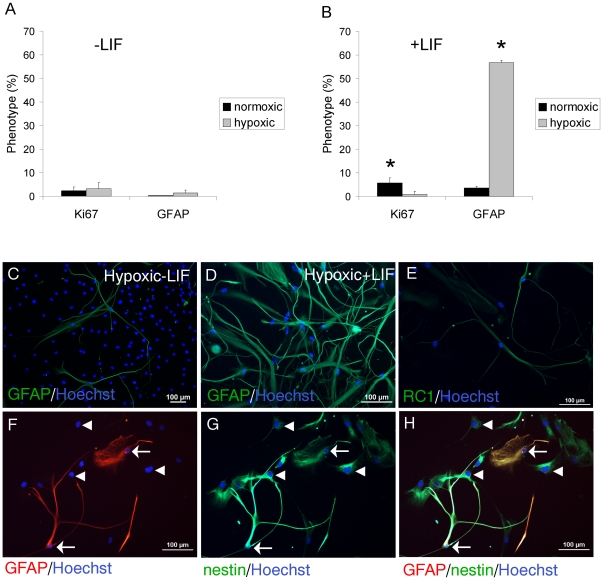
LIF enhances GFAP expression of adult human spinal cord NSPC in EGF/FGF2 culture. A, In the absence of LIF (3 cultures at 42, 55 and 66div), there is no significant difference in Ki67 or GFAP expression in normoxic or hypoxic conditions. B, In the presence of LIF (3 cultures at 35, 55, and 66div), there is a significant increase in Ki67 expression in normoxic versus hypoxic conditions (* p<0.025). LIF significantly increases GFAP expression in hypoxia (** p<0.001). C, GFAP^+^ NSPC in hypoxic culture in the absence of LIF. D, There are more GFAP^+^ cells in hypoxia + LIF. E, RC1^+^ NSPC (39div) in normoxic conditions. F, GFAP^+^ cells (arrows) are double-labelled with nestin (G) as shown in the merged panel (H). Arrows show GFAP^+/^nestin^+^cells. Arrowheads show GFAP^-/^nestin^+^cells.

The addition of 10ug/ml LIF resulted in a 2.5-fold increase in proliferation of NSPC in normoxia (2.3±1.6% Ki67 with no LIF vs. 5.7±2.1% Ki67 +LIF) (Fig. 4A,B), and a 7-fold increase in NSPC proliferation in normoxia relative to hypoxia (+LIF: 5.7±2.1% Ki67 in normoxia vs. 0.8±0.9% in hypoxia) (Fig. 4B). There was no significant effect of LIF on Ki67 expression in hypoxia, and thus the proliferative effect of LIF is attenuated in hypoxia. Hypoxia alone also had no significant effect on NSPC proliferation or GFAP expression (Fig. 4A). Most dramatically, LIF significantly increased GFAP expression (16-fold) in hypoxic conditions compared to normoxia (+LIF: 56.7±0.9% GFAP in hypoxia vs. 3.5±0.6% in normoxia) (Fig. 4B graph, Fig. 4C-D immunostaining). These GFAP^+^ NSPC also co-expressed nestin, as shown with double-label immunostaining in Fig. 4F-H. However, not all nestin^+^ cells co-expressed for GFAP, indicating no cross-reactivity of the GFAP antibody with nestin (Fig. 4F-H). This suggests that the LIF induced increase in GFAP expression promoted a radial glial phenotype. This was also suggested by RC1 immunostaining of some of the cultured cells (Fig. 4E). Similar staining was observed with 3CB2, another marker for radial glial cells. In addition, we found that neurospheres formed in either hypoxia or normoxia in the presence or absence of LIF, suggesting that neither LIF nor hypoxia are necessary for neurosphere formation.

### Adult human spinal cord NSPC are multipotent and directed differentiation can be promoted with exogenous factors

**Figure 5:**
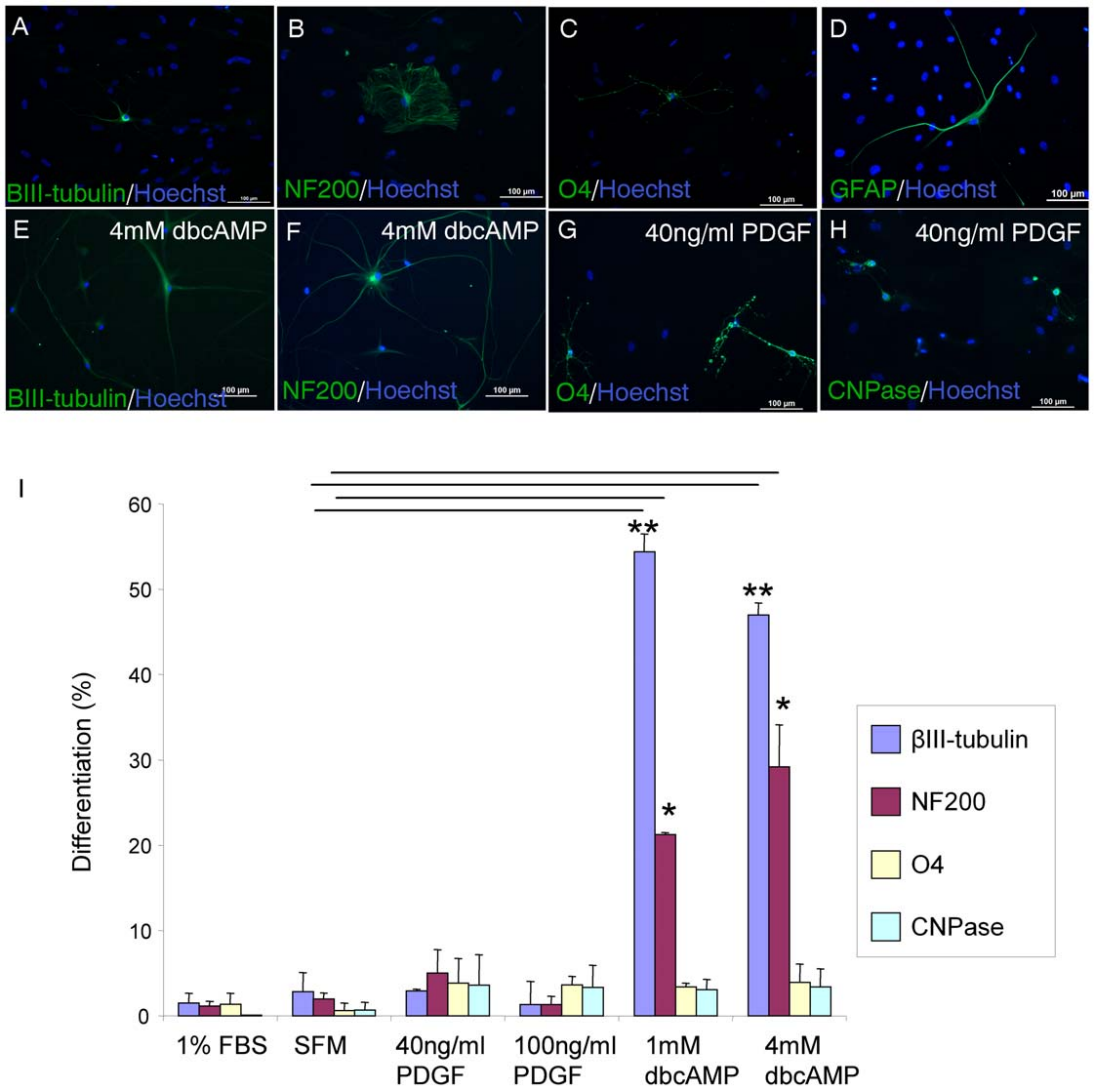
Adult human spinal cord NSPC are multipotent and directed differentiation can be promoted with exogenous factors. A-D, NPSC (from 3 cultures at 46, 58, and 72div) were plated in 1% FBS for 4wk and immunostained with cell type specific markers. NSPC expressed markers of neurons, βIII-tubulin (A) and NF200 (B), oligodendrocyte progenitor cell marker O4 (C), and GFAP (D) for astrocytes. The addition of dbcAMP increased the expression of βIII-tubulin (E) and NF200 (F). PDGF increased O4 (G) and CNPase (H) expression. I, Quantitation of % phenotype of NSPC after 4wk in dbcAMP, PDGF, and FBS or SFM controls. The expression of βIII-tubulin significantly increased in the presence of dbcAMP relative to SFM controls (** p<0.001). NF200 expression was also significantly increased compared to controls (* p<0.004). There was no significant difference in the quantitative expression of O4 and CNPase in any of the groups.

In differentiative conditions in the presence of serum for 4 weeks, adult human spinal cord NSPC demonstrated multipotency, differentiating into neurons, as shown with βIII-tubulin and NF200 staining (Fig. 5A-B), oligodendrocytes (O4, Fig. 5C), and astrocytes (GFAP, Fig. 5D). However, the percentage of NSPC differentiation was low, with 1.5±1.1% βIII-tubulin, 1.1±0.6% NF200, 1.3±1.2% O4, 0.1±0.08% CNPase and 0.2±0.07% GFAP expression in serum conditions. Also, cell proliferation and capacity to differentiate into oligodendrocytes decreased with increased time in culture. For example, the relative percentage of O4 positive cells decreased from 1.3% at 1 month in culture to 0.01% at 4 months in culture. We then investigated the capacity of exogenous factors to promote directed differentiation of adult human spinal cord NSPC (from 3 cultures at 46, 58, and 72div). PDGF enhanced oligodendrocyte progenitor proliferation and differentiation [54]-[56], and dibutyryl cyclic AMP (dbcAMP) which is a membrane permeable analog of cAMP enhanced neuronal differentiation of hippocampal progenitor cells and fetal rat striatal NSPC [57], [58]. Recently, dbcAMP promoted neuronal differentiation of adult rat brain NSPC [59]. We found that

 1 mM or 4 mM dbcAMP promoted neuronal differentiation of adult human spinal cord NSPC. With dbcAMP, NSPC showed increased βIII-tubulin and NF200 expression (Fig. 5E–F), and displayed enhanced neurite outgrowth and increased network formation (Fig. 5A–B, E–F). As shown in Fig. 5I, βIII-tubulin expression in 1 mM dbcAMP significantly increased by 19-fold relative to control (54.4±2.1% βIII-tubulin in dbcAMP vs. 2.8±2.2% in SFM). NF200 expression also significantly increased 15-fold relative to control (21.2±0.3% NF200 in dbcAMP vs. 1.9±0.7% in SFM). Fewer cells survived in wells incubated with dbcAMP suggesting selective death of non-neuronal progeny or survival of neuronal progenitor cells. PDGF-AA promoted oligodendrocyte differentiation as shown with increased O4 and CNPase immunostaining (Fig. 5G-H). Both PDGF and dbcAMP similarly increased O4 and CNPase immunoreactivity, but this was not statistically significant from control (Fig. 5I).

### Transplantation of adult human spinal cord NSPC into the injured rat spinal cord

**Figure 6:**
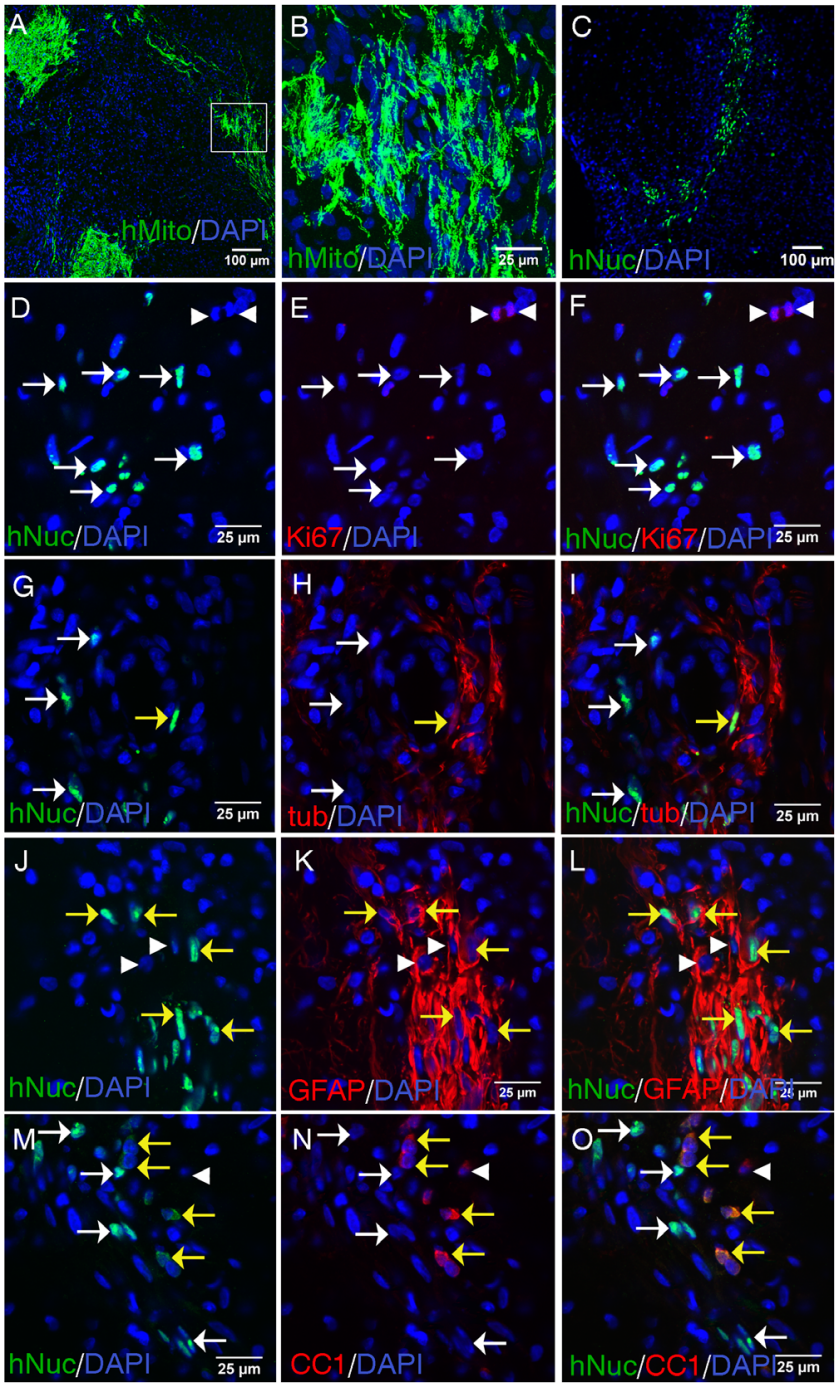
Transplantation of adult human spinal cord NSPC into the injured rat spinal cord. Adult human spinal cord NSPC were transplanted rostral and caudal to the lesion site at 1wk post-SCI. Rats were sacrificed at 1wk post-transplantation and tissue was sectioned in a parasagittal orientation. A, Low magnification fluorescent image showing transplanted cells (green) identified with human specific mitochondrial antigen (hMito). Tissue was stained with DAPI nuclear counterstain (blue). B, High magnification confocal image of boxed area showing hMito^+^ transplanted cells. C, Low magnification image showing transplanted cells (green) identified with human specific nuclear antigen (hNuc). D-F, Ki67^+^ (red) proliferating cells (arrowheads) adjacent to hNuc^+^ (green) transplanted cells. G-I, Transplanted NSPC expressing βIII-tubulin (tub); hNuc^+^/tub^+^ (yellow arrows); hNuc^+^/tub^-^ cells (white arrows). J-L, Transplanted NSPC expressing GFAP; hNuc^+^/GFAP^+^ (yellow arrows); hNuc^-^/GFAP^+^ (arrowheads). M-O, Transplanted NSPC expressing CC1; hNuc^+^/CC1^+^ (yellow arrows); hNuc^+^/CC1^-^ (white arrows); hNuc^-^/CC1^+^ (arrowheads).

Rats were injured with a moderate clip compression SCI, and 1wk later, NSPC were transplanted rostral and caudal to the lesion site. At 1wk post-transplantation, adult human spinal cord NSPC were identified with an antibody against human specific mitochondrial antigen (hMito) (Fig. 6A, confocal Fig 6B). Transplanted NSPC were also identified with human specific nuclear antigen (hNuc) (Fig. 6C), which was used for double-label immunostaining to identify the phenotypic fate of the grafted cells. Since the hNuc antibody labels only the nucleus of transplanted cells it is useful to use in combination with cell type-specific antibodies to assess double-labelling. In comparison, the hMito antibody labels the cellular processes, and showed extensive process extension from the transplanted cells (Fig. 6B). The average percentage of hNuc^+^ grafted cells surviving at 1wk post-transplantation was 7.6±4.72%, which is comparable to our previous studies with subacutely transplanted rat spinal cord NSPC into the injured cord [13], [17]. We did not observe hNuc^+^/Ki67^+^ cells (Fig. 6D-F), suggesting that the transplanted cells became post-mitotic. Ki67^+^ cells were often found adjacent to hNuc^+^ transplanted NSPC (Fig. 6F). In the injured rat spinal cord, transplanted human spinal cord NSPC expressed βIII-tubulin (Fig. 6G-I), GFAP (Fig. 6J-L), and CC1 (Fig. 6M-O), showing differentiation into neurons, astrocytes, and oligodendrocytes, respectively. Thus, adult human spinal cord NSPC survive following transplantation into the injured rat spinal cord and differentiated into neurons, astrocytes, and oligodendrocytes.

## Discussion

We showed that proliferating and self-renewing NSPC can be cultured from the adult human spinal cord from organ transplant donors, and can be passaged as an adherent monolayer in the presence of EGF and FGF2. Also, these neurospheres can form in suspension culture at clonal density when the NSPC are initially cultured as an adherent monolayer. Cells cultured from the adult human spinal cord under conditions described in the present study are a heterogenous population comprised of both stem and more restricted progenitor cells. To our knowledge, no other group has reported successful passaging and expansion of adult human spinal cord-derived NSPC and the transplantation of these cells. We refer to these cells as a mixed population of neural stem/progenitor cells since they display properties of self-renewing multipotent stem cells and more restricted progenitors.

Our initial attempts to culture these NSPC as neurospheres in suspension culture were unsuccessful, even with the addition of various factors such as LIF, BIT and hypoxic conditions, which previous work has shown to increase the expansion of NSPC [29], [33], [40], [42]–[45]. Culturing these cells as an adherent monolayer in EGF/FGF2 supplemented medium was effective in selecting and expanding the NSPC population. Primary culture from the adult human spinal cord is very heterogenous, consisting of various differentiated cells in addition to the precursor cells, myelin and cell debris. The mitogenic growth factors select for the precursor cells, and myelin and cell debris are removed with media changes in an adherent culture.

In EGF/FGF supplemented medium, these NSPC proliferate and primarily express markers of stem cells such as nestin and Sox2, and low levels of markers of more differentiated cells such as BIII-tubulin, O4, and GFAP. Although nestin and Sox2 are also expressed by reactive astrocytes, it is unlikely that the human spinal cord cultures are comprised of reactive astrocytes since they express low levels of GFAP. Also, the expression of nestin and Sox2 was observed early in culture by the first passage. This phenotypic expression pattern in proliferative culture conditions is consistent with our previous work with NSPC derived from the adult rat spinal cord [11], [13], and with other studies with NSPC from the rodent and human fetal spinal cord [20], [31], [60], [61]. In hypoxia, we found no significant difference in human spinal cord NPSC proliferation, which is contrary to previous studies [42]–[45] perhaps because the other studies used neurospheres from embryonic or fetal cells. Also, in the present study, the adult human spinal cord NSPC were maintained and exposed to hypoxia from the time of isolation. Consistent with our findings, van der Kooy's group found that primary colonies derived from embryonic and adult mouse brain NSPC had little response to hypoxia [62]. Increased colony formation in hypoxia was only seen after one or more passages in normoxia [62].

LIF is important for self-renewal of mouse embryonic stem cells and has increased the rate of proliferation of fetal neural stem cells [29], [39]–[41]. Consistent with these studies, LIF significantly increased the proliferation of adult human spinal cord NSPC in normoxia. However, this effect was not apparent in hypoxia suggesting that low oxygen abrogates the effects of LIF. LIF also significantly enhanced GFAP expression in hypoxia, and these GFAP cells co-expressed nestin. Similarly, LIF was shown to upregulate GFAP in nestin positive cells cultured from human fetal cortex [40], and LIF and BMP4 were shown to act synergistically to induce astrocyte differentiation from human fetal spinal cord precursors [63]. Other studies have reported the co-expression of nestin and GFAP in NSPC [22], [47], [64], [65], suggesting that these cells represent radial glial cells, which are recognized as multipotent neural stem cells [66]–[68].

We also showed that adult human spinal cord NSPC are multipotent, generating neurons, astrocytes, and oligodendrocytes. However, unlike NSPC from the adult rat spinal cord, human spinal cord NSPC do not preferentially differentiate into oligodendrocytes, suggesting a species specific difference in phenotypic potential. In terms of donor age, human spinal cord NSPC derived from the younger donor yielded a higher percentage of neuronal, astrocyte and oligodendrocyte differentiation, although this difference was not marked. Overall, the capacity for NSPC differentiation diminished with increased time in culture, consistent with previous reports [18], [20], [23], [26], [64], [69].

The present study also showed the response of adult human spinal cord NSPC to exogenous factors which promoted selective differentiation. DbcAMP enhanced neuronal differentiation, similar to previous reports with fetal and adult rat brain NSPC [57]–[59]. DbcAMP markedly enhanced morphological differentiation of human spinal cord NSPC and their expression of neuronal markers. Previously, we showed that dbcAMP is one of the most effective factors in inducing neuronal differentiation of adult rat brain NSPC [59]. Consistent with our earlier study, the effects of dbcAMP on the in vitro differentiation of human spinal cord NSPC were not proliferative, suggesting a selective survival of neuronal progenitor cells or alternatively, cell death of non-neuronal progenitor cells. We have not examined whether the neurons formed from adult human NSPC are interneurons or motorneurons. Guo et al., showed that a combination of factors including Shh, retinoic acid, FGF2, and vitronectin promoted the differentiation of a fetal human spinal cord stem cell line to motorneurons [70]. Also consistent with our earlier study [59], an increase in the percentage of NSPC expressing oligodendrocyte markers in response to dbcAMP was observed. This suggests pleiotropic actions of dbcAMP on NSPC differentiation which are not species-specific. PDGF-AA similarly increased oligodendrocyte differentiation, but this was not statistically significant. This is consistent with other reports describing limited differentiation of human fetal NSPC in response to PDGF [20], [27], [29], [31]. Fewer oligodendrocytes were generated from human embryonic spinal cord neural precursors relative to rodent cells [20]. We plan to examine other factors to promote oligodendrocyte differentiation.

The adult human spinal cord NSPC survived after subacute transplantation into the injured rat spinal cord, and retained the capacity to differentiate into neurons, astrocytes, and oligodendrocytes. Also, the xenografted human NSPC did not express Ki67 1 week post-transplantation, suggesting they became post-mitotic. The present study provides a better understanding of specific factors and conditions that promote adult human NSPC expansion and differentiation. Also, factors which promote selective differentiation of human NPSC may be used for the pre-differentiation of these cells prior to transplantation or the factors may be administered after grafting to promote targeted differentiation in vivo. Next, we plan to examine functional recovery following transplantation of these cells in experimental SCI. This is the first study to show that self-renewing multipotent NSPC can be isolated and passaged from adult human spinal cords from organ transplant donors, and that these cells survive and differentiate following transplantation into SCI rats. It is possible that these cells may be useful for transplantation in humans with SCI or other diseases of the spinal cord. The ease with which neuronal differentiation can be induced is especially attractive.
